# Optimizing Ridge Augmentation With AI‐Generated Models: A Case Report With Technical Note

**DOI:** 10.1002/ccr3.71685

**Published:** 2025-12-19

**Authors:** Vasilios Alevizakos, Stephan Knörzer, Roman Krammer, Marcus Schiller, Constantin von See

**Affiliations:** ^1^ Research Centre for Digital Technologies in Dentistry and CAD/CAM Danube Private University Krems Austria; ^2^ Oral Surgery Private Practice—Dr. Krammer and Colleagues Amberg Germany; ^3^ Oral and Maxillofacial Surgery Medical School Hannover Hanover Germany

**Keywords:** alveolar ridge augmentation, artificial intelligence, cone beam computed tomography, dental implants, three‐dimensional printing

## Abstract

AI‐generated 3D models in ridge augmentation improve surgical accuracy, reduce bone exposure, and enhance patient understanding through visual planning. They support, but do not replace, clinical expertise. Ongoing validation, staff training, and ethical oversight are essential to ensure safe, effective, and equitable implementation in daily practice.

## 1 Introduction

Artificial intelligence (AI) is emerging as a transformative tool in oral surgery, offering innovative solutions to long‐standing clinical challenges [1]. Still an emerging technology, AI shows strong potential for addressing unmet clinical needs. Traditional ridge augmentation methods often suffer from imprecise cortical plate adaptation, radiographic artifacts, and prolonged intraoral manipulation, increasing surgical time and complication risks [2, 3]. Manual planning methods are also prone to variability depending on clinician expertise, which can compromise reproducibility and treatment predictability.

AI can overcome these limitations by enhancing diagnostic imaging and surgical planning. Using machine learning techniques such as convolutional neural networks (CNNs), AI enables high‐precision analysis of complex imaging data, improving feature detection and artifact reduction [4, 5, 6].

Recent studies show that AI‐based workflows improve defect analysis, graft adaptation accuracy, and reduce chairside time, although learning curves and defect variability remain challenges [7, 8, 9]. In ridge augmentation, AI‐driven DICOM processing allows optimized 3D model generation by reducing metal artifacts, supporting accurate bony assessment and precise cortical plate adaptation [10, 11].

These capabilities contribute to improved efficiency, decision‐making, and reduced intraoperative time [12]. In this case, an AI‐generated model enhanced bone block adaptation during surgery [13, 14].

Despite these benefits, ethical concerns such as data privacy, algorithm transparency, and bias remain critical [15]. While conventional methods remain effective in expert hands, AI should be viewed as an adjunct that enhances precision, standardizes workflows, reduces surgical risk, and supports rather than replaces clinical judgment [16, 17, 18].

## 2 Materials and Methods

### 2.1 Consent

All procedures performed in studies involving human participants were conducted in accordance with the ethical standards of the institutional and/or national research committee and with the 1964 Helsinki Declaration and its later amendments or comparable ethical standards. Written informed consent was obtained from the patient for publication of this case report and any accompanying images.

### 2.2 Case History/Examination

**FIGURE 1:**
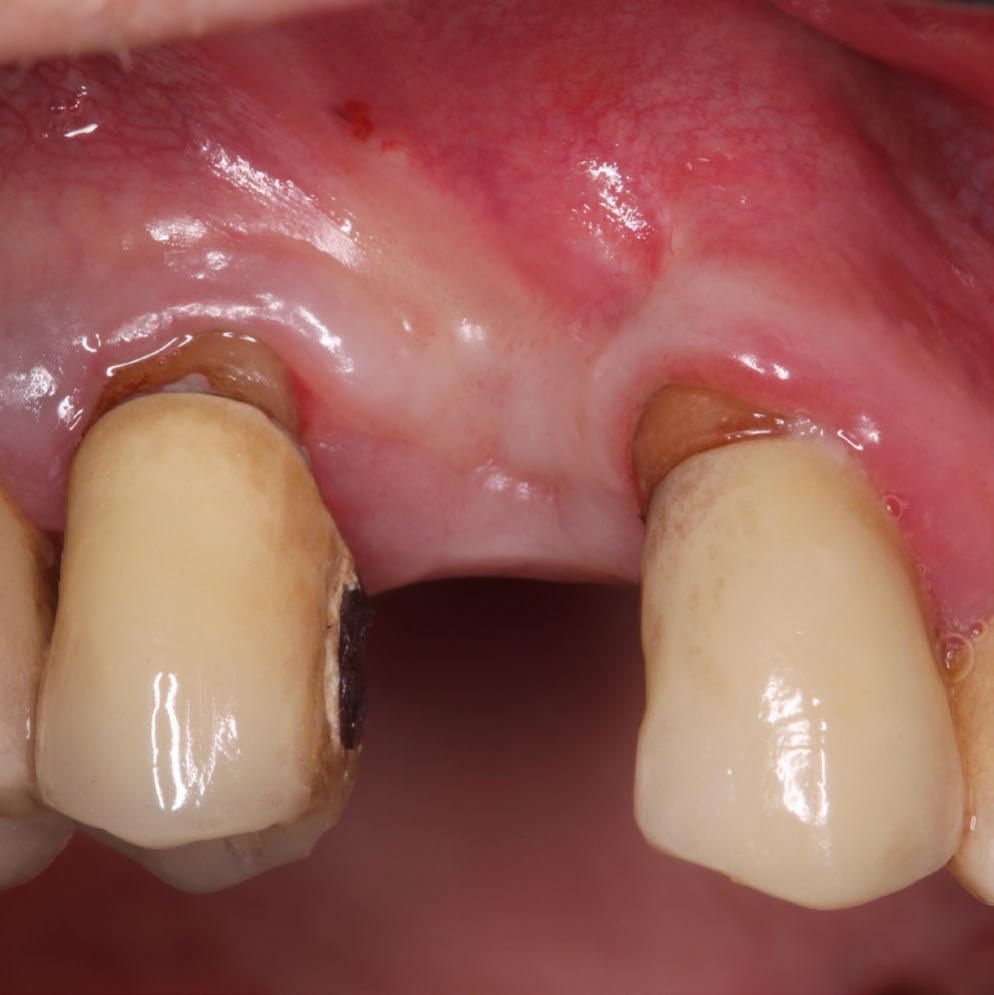
Facial photography of the tooth gap in Region 14. The alveolar ridge has receded vertically.

**FIGURE 2:**
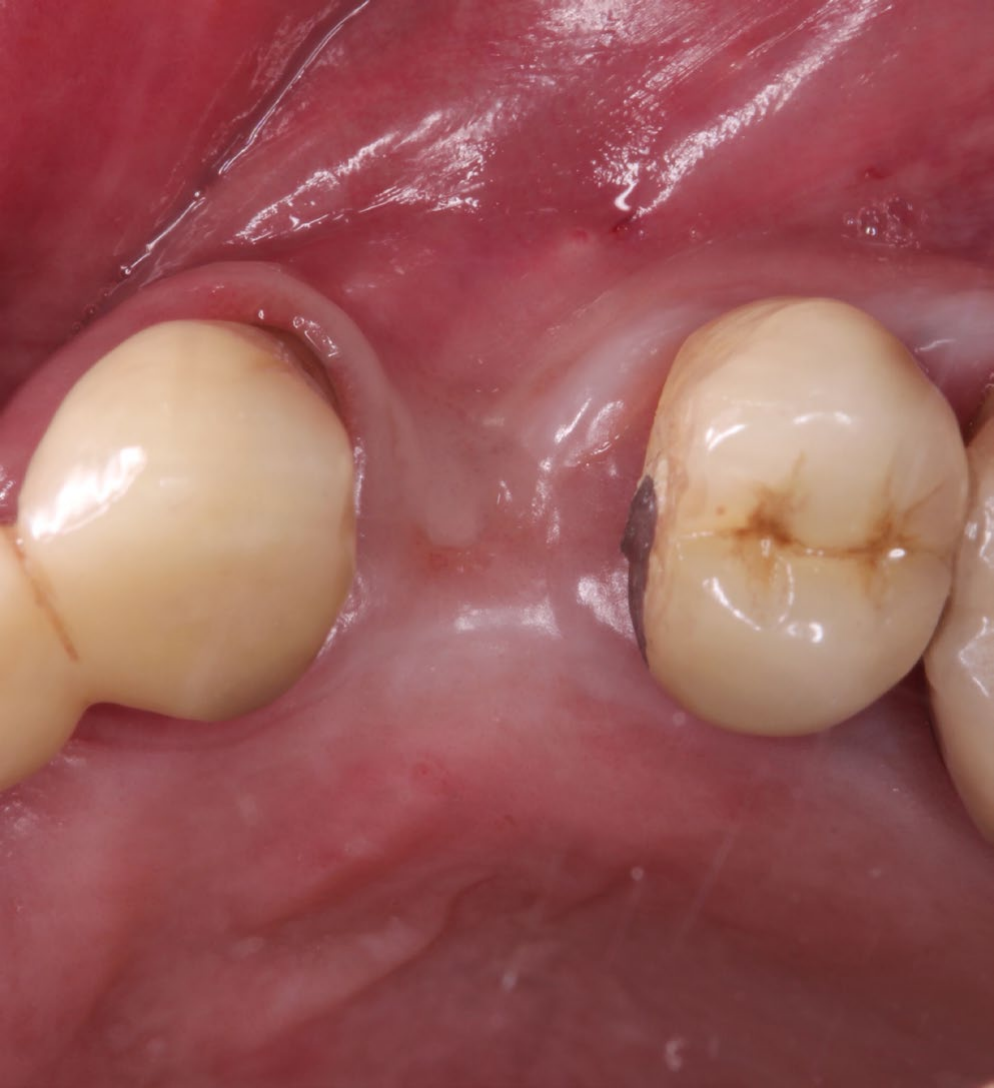
Occlusal photography of the tooth gap in Region 14. The alveolar ridge has receded horizontally.

**FIGURE 3:**
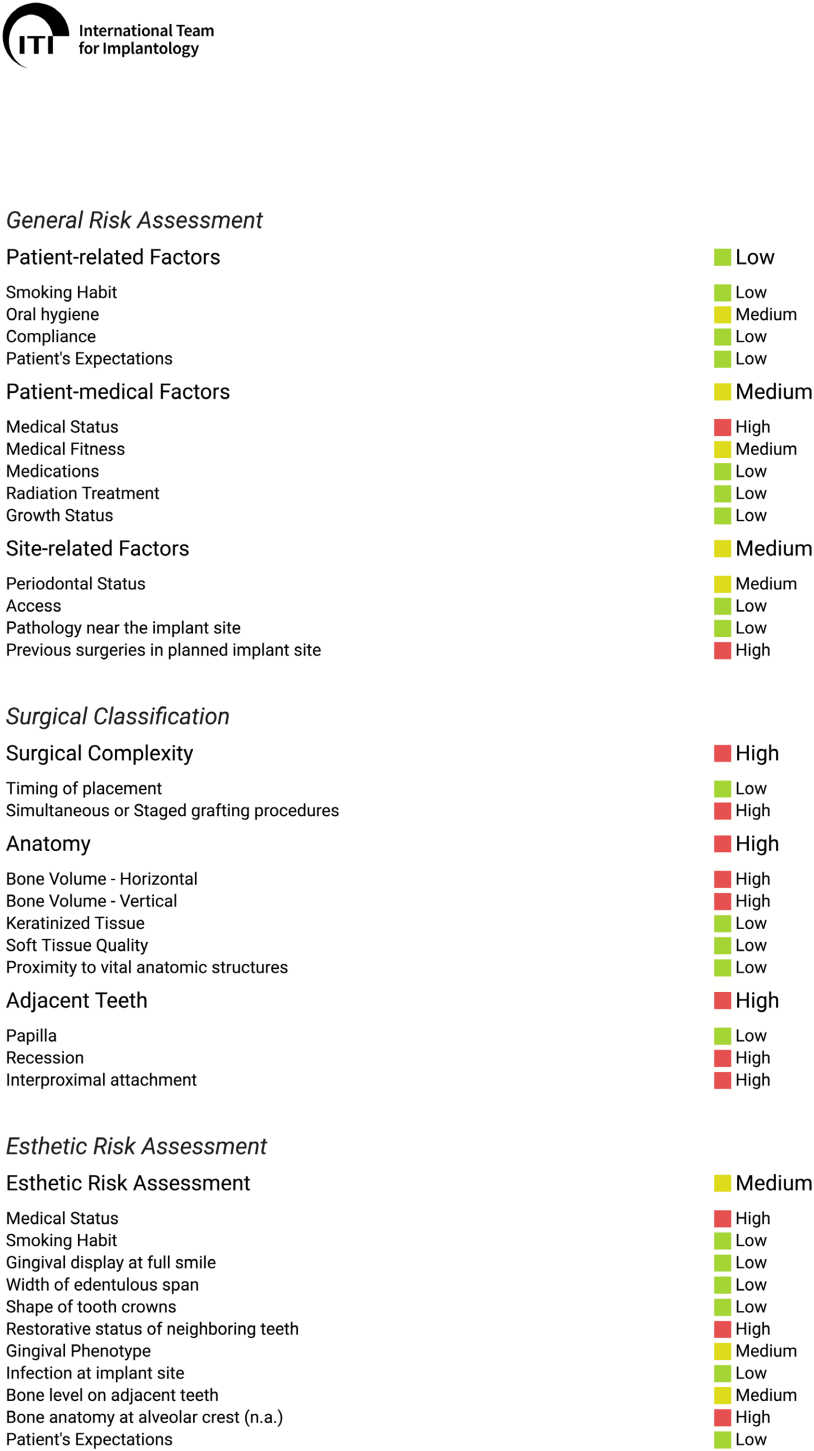
Risk assessment of the planned intervention. In the general risk assessment, the patient‐related factors show low risk. The patient‐medical and site‐related factors show medium risk. Regarding the surgical classification, it shows to be complex in terms of surgical complexity, anatomy, and adjacent teeth. The aesthetic risk assessment shows medium risk.

A 69‐year‐old male presented with a solitary gap in the anterior maxillary posterior region due to the absence of Tooth 14 and requested implant‐based rehabilitation for aesthetic reasons. Apart from hypertension and a known nickel allergy, no relevant systemic diseases were present. Clinical examination revealed combined vertical and horizontal bone loss in Region 14 (Figures 1 and 2). The patient was comprehensively informed about the surgical procedure and risks (Figure 3).

Patient selection for the AI‐assisted workflow was based on the high aesthetic demand, pronounced anatomical limitations, and the need for customized material planning due to the nickel allergy. The complex defect morphology made conventional planning challenging and justified the use of AI‐supported digital planning.

AI was applied to optimize functional and aesthetic treatment outcomes by enabling precise, individualized planning. Its predictive capabilities allowed simulation of different treatment scenarios, supporting a personalized, outcome‐focused approach for this demanding clinical situation.

### 2.3 Imaging and AI‐Based Planning

**FIGURE 4:**
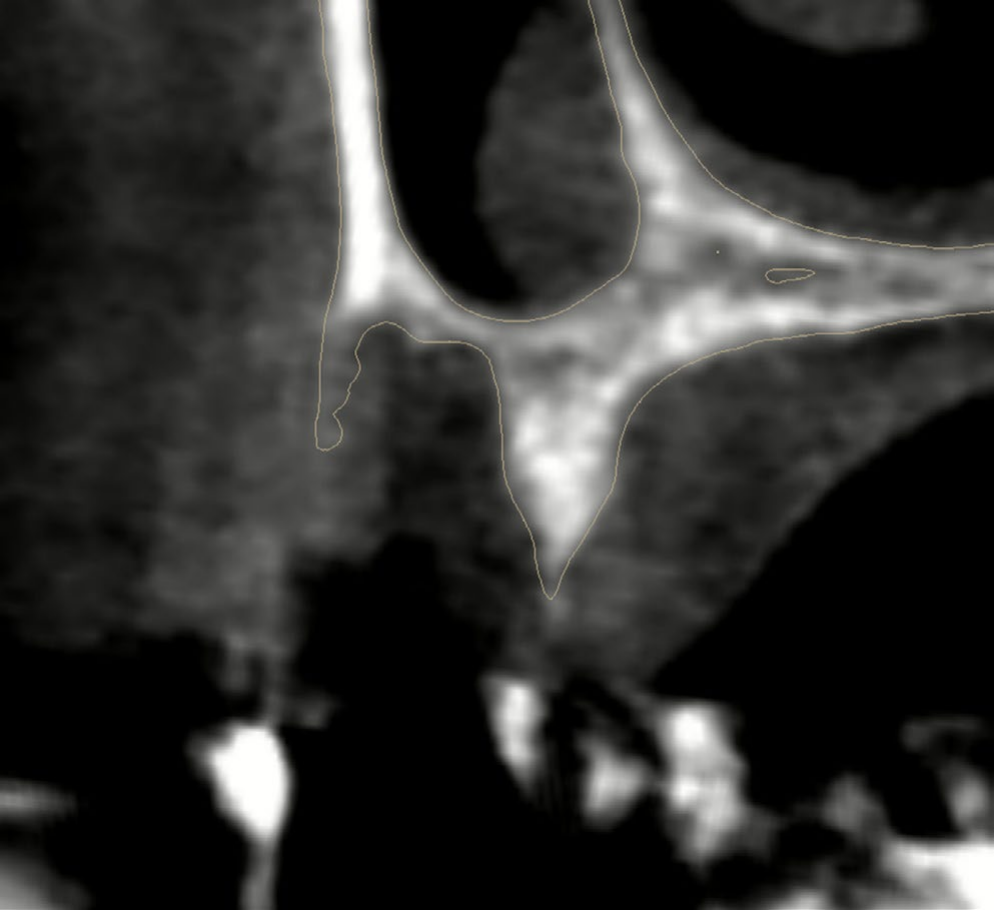
Cross section of the alveolar socket in the CBCT scan showing the vertical defect.

For diagnostic evaluation, a cone beam computed tomography (CBCT) scan was performed (4.4 mA, 90 kVp; PaX‐Uni 3D, Vatech, South Korea), revealing a significant buccal wall defect in the alveolar socket (Figure 4).

**FIGURE 5:**
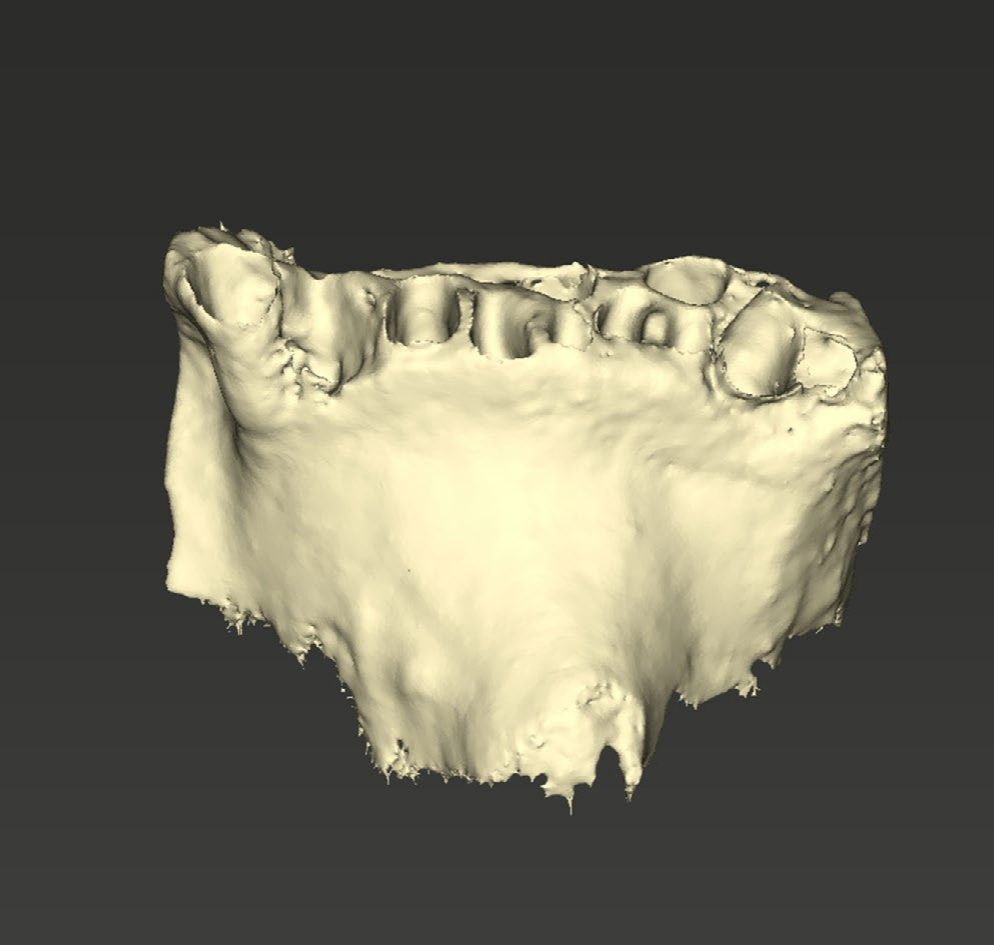
AI‐generated model of the jaw from lateral view.

**FIGURE 6:**
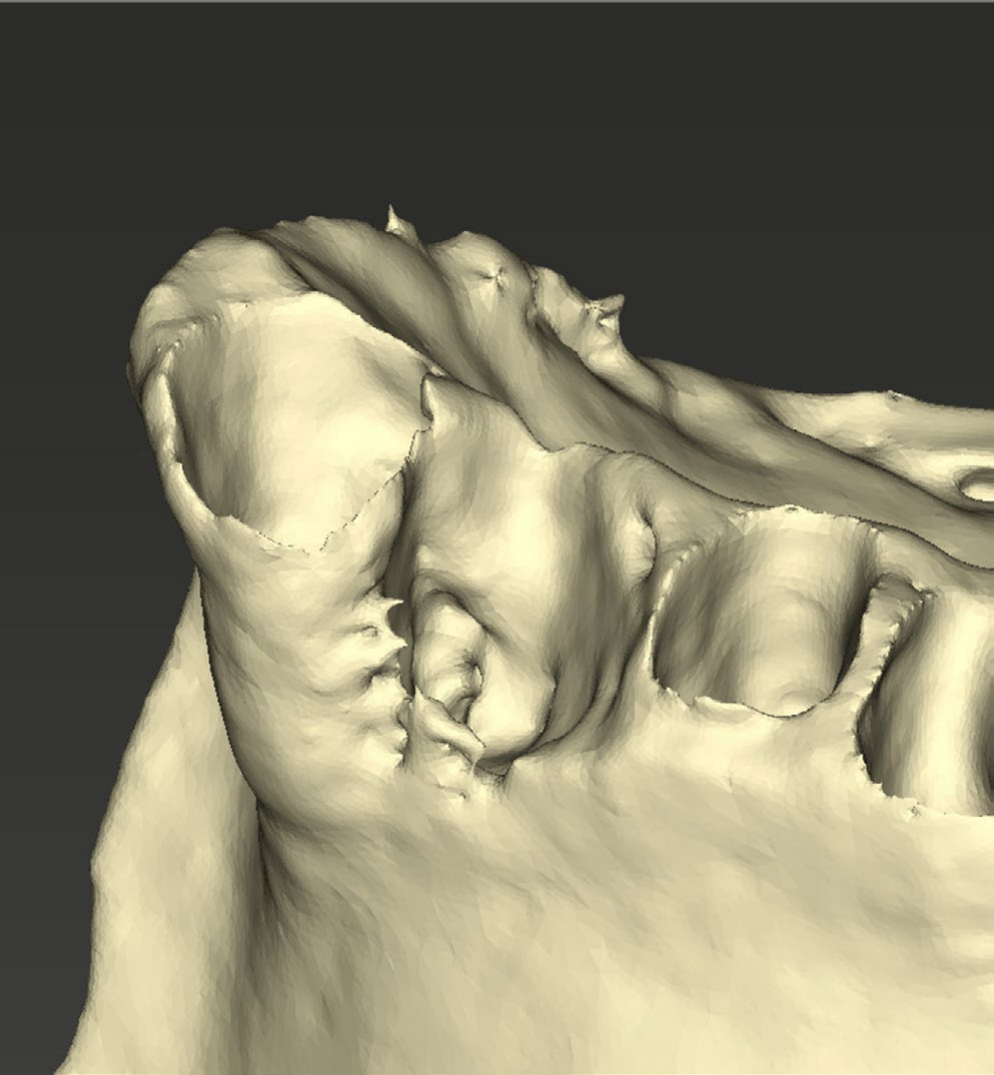
Close‐up of the AI‐generated model of the jaw from a lateral view showing the bony defect of the alveolar socket.

AI‐based planning was conducted using CoDiagnostiX (v10.7; Dental Wings GmbH, Germany), selected for its CE certification, established clinical use, AI‐driven artifact correction, and STL export capability for 3D printing. The CBCT data containing artifacts were processed using CNN–based algorithms trained on large radiographic datasets. Although high accuracy and reproducibility have been reported, dependency on image quality and potential misclassification remain recognized limitations (Figures 5 and 6).

**FIGURE 7:**
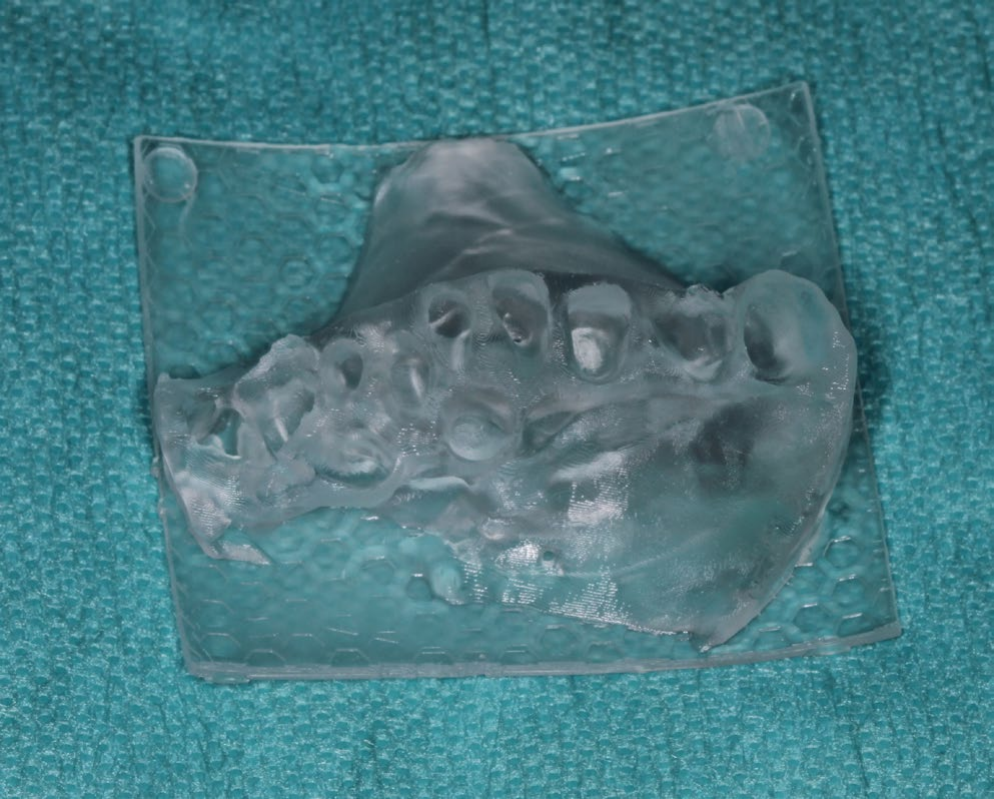
3D‐printed jaw model.

After artifact correction, automatic segmentation of teeth and bone was performed and verified by the clinical team to ensure accuracy. A clean 3D model was generated, exported as an STL file, and 3D printed (P20+, P PRO SURGICAL GUIDE; Straumann AG, Switzerland) (Figure 7). The sterilized model (134°C, 2.1 bar; Vacuklav40‐B, MELAG, Germany) was used intraoperatively for precise cortical plate adaptation.

### 2.4 Materials Used

For transparency and reproducibility, the following materials were used in the procedure:

- Allogeneic cortical plate: maxgraft cortico, botiss biomaterials; GmbH, Zossen, Germany.
- Bone graft substitute: cerabone, 0.5–1.0 mm, botiss biomaterials; GmbH, Zossen, Germany.
- Fixation screws: steel microscrews, Ø 1.0 mm × 13 mm; Ustomed Instrumente GmbH, Tuttlingen, Germany.
- Barrier membrane: OSSIX PLUS, 25 × 30 mm; REGEDENT GmbH, Dettelbach, Germany.
- Sutures: nonabsorbable polyamide monofilament, size 5‐0, B.Braun, Melsungen, Germany.
- AI planning software: CoDiagnostiX v10.7; Dental Wings GmbH, Chemnitz, Germany.
- 3D printing resin: P PRO SURGICAL GUIDE; Institut Straumann AG, Basel, Switzerland.

### 2.5 Surgery

The patient received a 2 g dose of a penicillin‐based antibiotic orally (augmentin [amoxicillin 875 mg/clavulanic acid 125 mg]; GlaxoSmithKline GmbH & Co. KG, Munich, Germany) as a perioperative antibiotic shield, administered 60 min prior to the surgery. Additionally, for temporary reduction of bacterial load, the patient rinsed their oral cavity with 10 mL of 0.2% chlorhexidine bis(D‐gluconate) solution for 1 min (Chlorhexamed FORTE alcohol‐free 0.2%; GlaxoSmithKline GmbH & Co. KG, Munich, Germany).

The patient was aseptically draped and received 1.7 mL of epinephrine‐enhanced articaine hydrochloride (Ultracain D‐S forte 1:100,000; Septodont GmbH, Niederkassel, Germany) for vestibular and oral infiltration anesthesia at Region 14.

**FIGURE 8:**
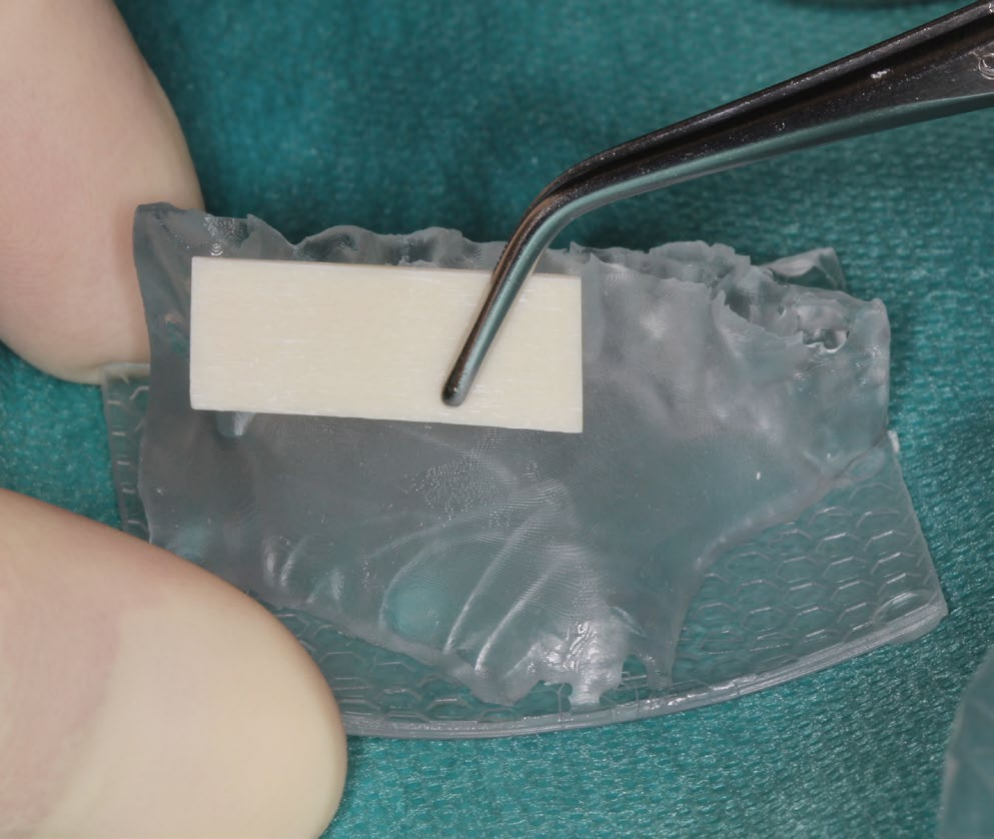
First adjustment of the allogenic cortical plate at the printed jaw model from lateral view.

**FIGURE 9:**
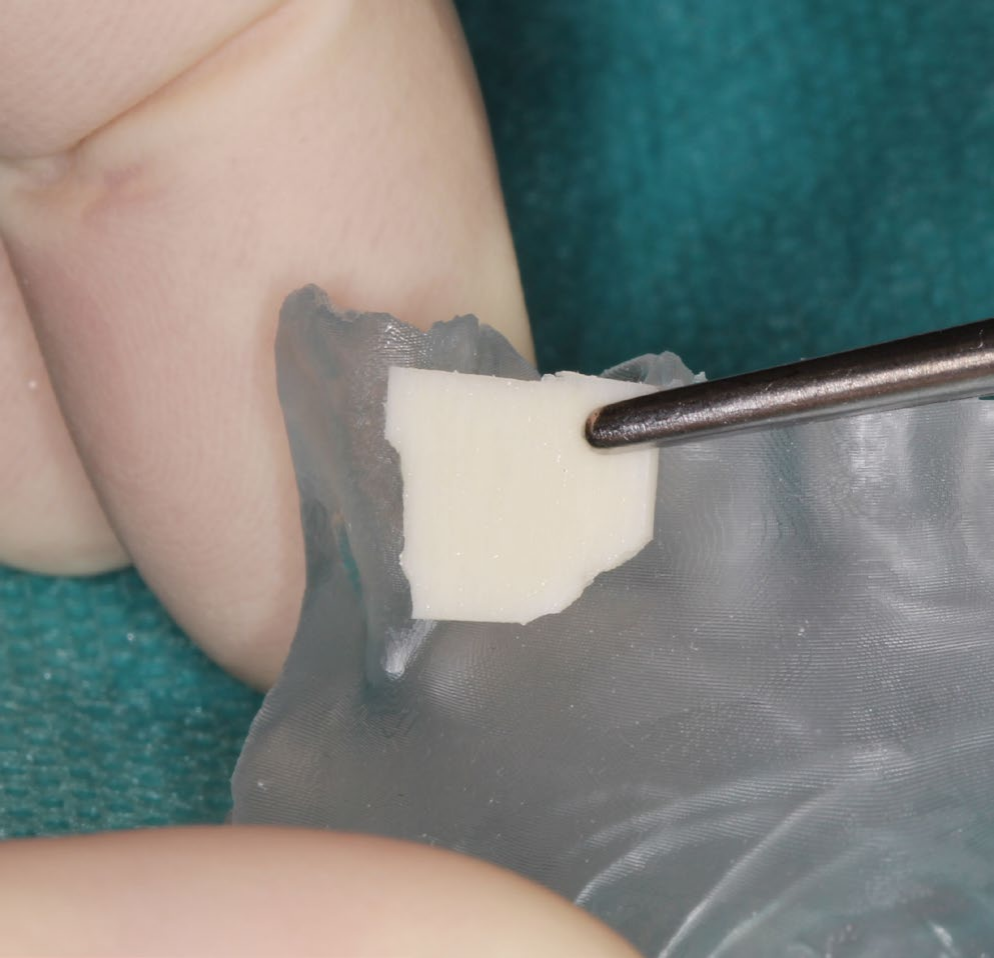
Testing the fitting of the adapted plate on the defect from the lateral view.

Prior to flap elevation, the allogeneic cortical plate (maxgraft cortico, botiss biomaterials; GmbH, Zossen, Germany) was extraorally adapted to the sterilized AI‐generated model (Figures 8 and 9). This step required approximately 6 min, which contrasts with the 20–25 min typically required for intraoral plate adaptation in similar defects using conventional methods, as reported in previous literature. This time reduction minimized intraoral bone exposure, thereby lowering the risk of flap contraction and associated complications.

An incision was made, starting intrasulcularly at Tooth 13 as a crestal incision in the region of the gap, and concluding intrasulcularly at Tooth 15 with vertical relief incisions mesially at Region 13 and distally at Region 15. The bony defect was exposed by elevating a mucoperiosteal flap.

**FIGURE 10:**
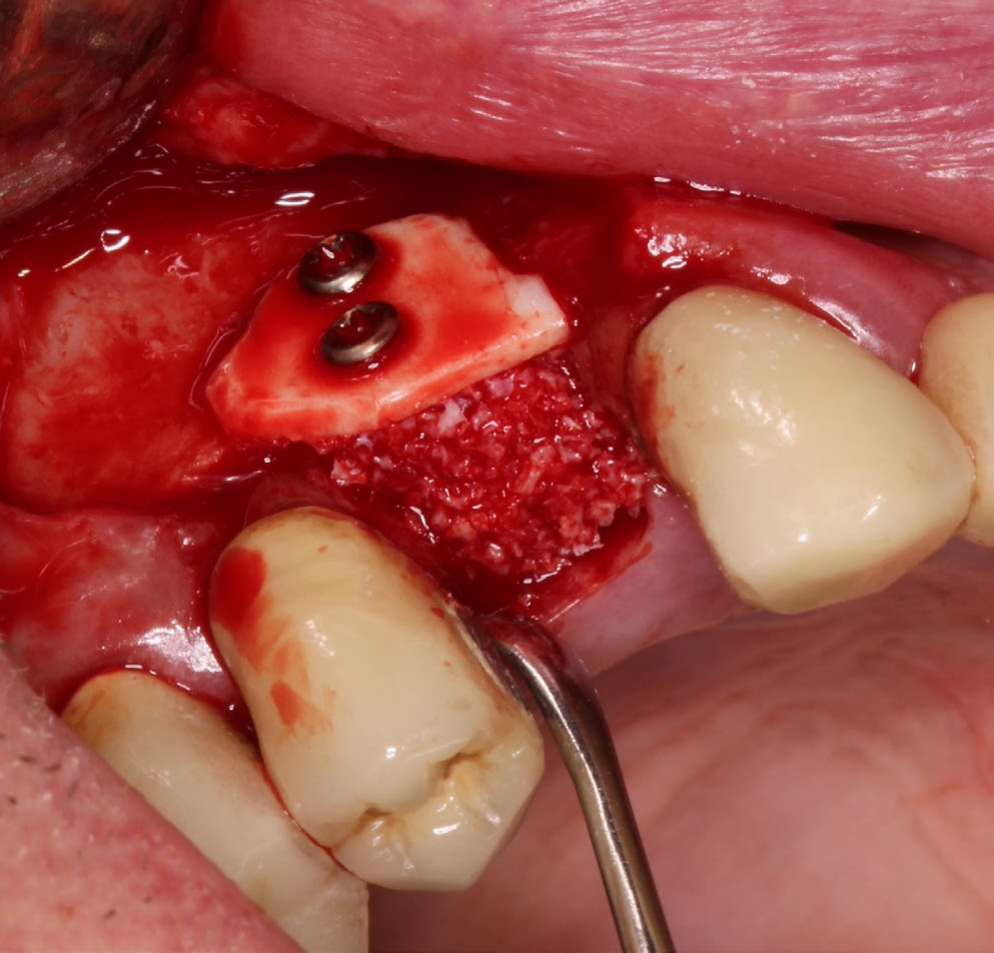
The buccal wall is restored by the cortical plate and the socket filled with bovine bone grafting material.

**FIGURE 11:**
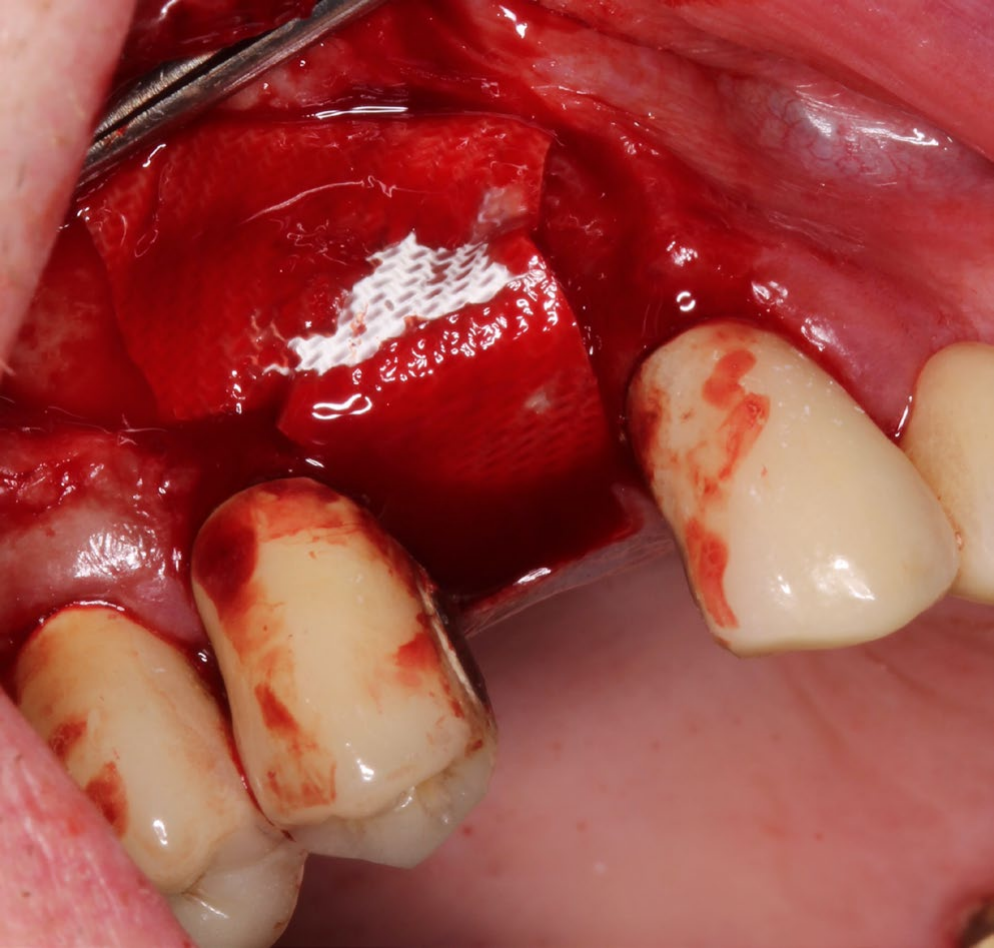
The augmented ridge is covered by a collagenous membrane.

**FIGURE 12:**
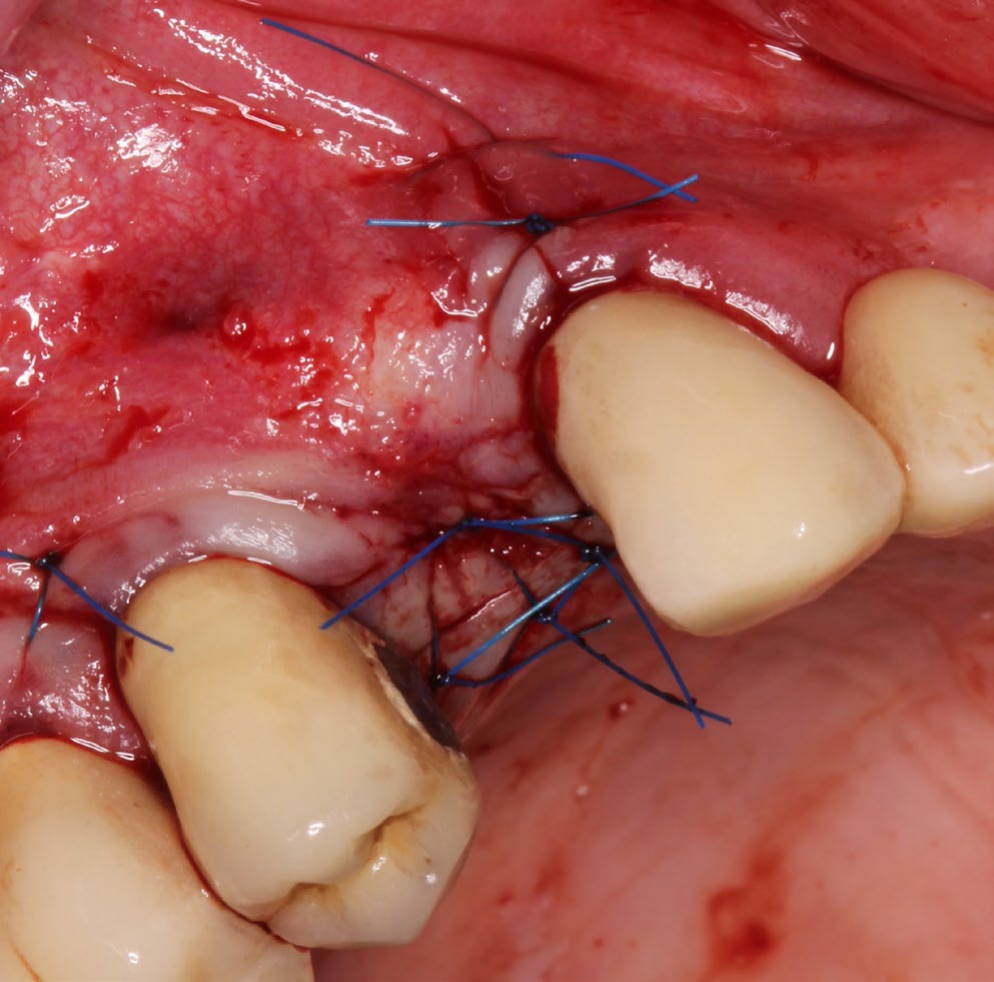
The mucoperiosteal flap was sutured with nonabsorbable polyamide monofilament sutures after periosteal slitting, ensuring a tension‐free and saliva‐proof closure.

The adapted cortical plate was secured with two microscrews (cross, steel, Ø 1.0 mm, 13 mm; Ustomed Instrumente GmbH & Co. KG, Tuttlingen, Germany), and the alveolar socket was filled with bovine bone grafting material (cerabone, 0.5–1.0 mm, 1.0 mL, botiss biomaterials; GmbH, Zossen, Germany) (Figure 10). The bone augmentation was then covered with a collagen membrane (OSSIX PLUS, 25 mm × 30 mm; REGEDENT GmbH, Dettelbach, Germany), and the mucoperiosteal flap was sutured with nonabsorbable polyamide monofilament sutures after periosteal slitting, ensuring a tension‐free and saliva‐proof closure (Figures 11 and 12).

**FIGURE 13:**
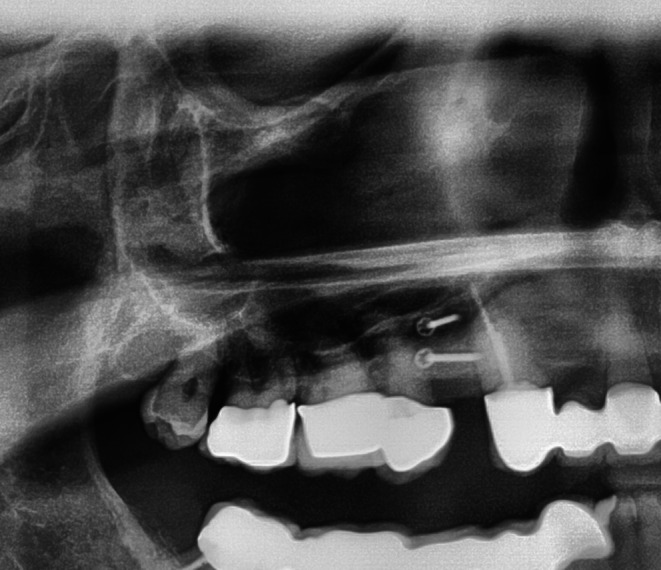
The postoperative partial panoramic X‐ray shows the augmented ridge and screw fixation.

Immediately postoperatively, an orthopantomogram was taken (12 mA, 74 kVp; PaX‐Uni 3D; Vatech, Gyeonggi‐do, South Korea) (Figure 13).

While the AI‐generated plan facilitated accurate adaptation and reduced intraoral time, potential risks include overreliance on the model without adequate clinical validation, inaccuracies in segmentation leading to misfit of the graft, and the need for manual intraoral adjustments if discrepancies arise. In this case, no intraoperative complications occurred, but these limitations should be acknowledged for clinical safety.

## 3 Results

Both the extraoral processing of the cortical plate on the model and the surgery proceeded without complications. No fracture of the cortical plate or injury to adjacent anatomical structures occurred. The plate was stably fixed with osteosynthesis screws, and the bony defect was successfully reconstructed.

Adaptation on the AI‐generated model required only 6 min, compared with the usual 20–25 min for intraoral adaptation, resulting in reduced bone exposure and potentially improved healing. Postoperatively, healing was uneventful with stable soft tissue closure and no infection or dehiscence, although long‐term outcomes remain unavailable.

## 4 Discussion

In this case presentation, we demonstrated the use of AI in ridge augmentation with an allogeneic cortical plate and bovine bone substitute. A precise 3D jaw model was generated from CBCT‐derived DICOM data and 3D printed, enabling accurate extraoral adaptation of the cortical plate in only 6 min, completely eliminating intraoral modifications.

AI has already shown promise in radiology and pathohistology, and its potential to improve precision and efficiency in oral surgery is significant. Our primary goal was to reduce intraoral bone exposure time, as prolonged exposure can impair healing and negatively affect soft tissue elasticity and flap management [14, 17, 18]. Delayed flap reattachment increases the risk of tension, slitting, bleeding, swelling, and postoperative discomfort [15, 19].

Recent studies support AI‐generated models for improved graft adaptation and reduced intraoral time, although challenges remain in workflow standardization, diverse defect morphologies, and learning curves. Ethical and regulatory aspects are critical, particularly concerning transparency, accountability, and patient data security. While CE‐marked tools such as CoDiagnostiX are established, continuous auditing is essential to ensure safety and reproducibility [7, 9, 20].

Technically, AI‐based segmentation is not error‐free and depends on CBCT quality. Risks include misclassification and discrepancies between virtual and intraoral anatomy. In this case, manual verification mitigated these risks; however, overreliance on AI must be avoided, and future systems should include real‐time error detection and clinician override functions [21].

Generalizability remains limited, as outcomes may vary with defect morphology, anatomy, and resource availability. Financial investment in software, hardware, and training may also restrict access and contribute to healthcare disparities [22].

AI should function as a decision‐support tool rather than replace clinical judgment, particularly for less experienced clinicians [23].

Finally, AI will influence interdisciplinary collaboration and patient communication. While visualization may improve patient confidence, transparency regarding data use and algorithmic decision‐making is essential to maintain trust [24].

Future research should include large‐cohort studies to compare AI‐assisted and conventional ridge augmentation regarding efficiency, complication rates, and long‐term outcomes. Continuous training is essential to ensure safe and effective use of AI systems. Further integration with augmented reality, robotics, and navigation may expand clinical applications.

In summary, AI offers clear potential to reduce procedure time and improve surgical precision and patient comfort. However, risks, costs, training demands, and ethical aspects must be carefully addressed before routine clinical implementation.

## 5 Limitations

This case report has important limitations. As a single‐patient study, the findings cannot be generalized, and different defect morphologies and patient profiles were not evaluated. The lack of long‐term follow‐up restricts conclusions regarding graft stability, bone integration, and long‐term implant outcomes.

Although CoDiagnostiX was selected for its CE certification and widespread use, its accuracy depends on CBCT image quality and is susceptible to segmentation and artefact‐related errors. AI in this workflow is not autonomous and requires continuous human oversight to avoid overreliance.

The study also lacked quantitative evaluation of algorithm performance and did not assess costs, training demands, or infrastructure requirements, potentially limiting accessibility. Larger, controlled studies are needed to validate clinical reliability and scalability.

## 6 Conclusion

AI‐assisted ridge augmentation in this case report improved surgical precision, workflow efficiency, and patient experience. AI‐generated 3D models enabled precise extraoral graft adaptation, reducing intraoral manipulation and bone exposure, which may lower postoperative complications and improve flap management.

AI also enhanced patient understanding through clearer preoperative visualization, increasing confidence. However, challenges remain regarding data security, algorithm transparency, and informed consent. The surgeon's role remains central, with AI serving as a decision‐support tool rather than a replacement.

From a systemic perspective, regulatory oversight, data protection, and continuous validation are essential. Future research should address scalability, auditing, and cost–benefit aspects to ensure equitable access. Overall, AI‐assisted planning is promising but requires careful ethical, technical, and economic consideration.

## Author Contributions

**Vasilios Alevizakos:** conceptualization, methodology, writing – original draft. **Stephan Knörzer:** data curation, investigation. **Roman Krammer:** data curation, resources. **Marcus Schiller:** writing – review and editing. **Constantin von See:** supervision.

## Funding

The authors have nothing to report.

## Conflicts of Interest

The authors declare no conflicts of interest.

## Data Availability

The data that support the findings of this study are available from the corresponding author upon reasonable request.
